# Maternal knowledge on early childhood caries and barriers to seek dental treatment in Jordan

**DOI:** 10.1007/s40368-020-00576-0

**Published:** 2020-11-18

**Authors:** A. BaniHani, J. Tahmassebi, F. Zawaideh

**Affiliations:** 1 School of Dentistry/Faculty of Medicine and Health, NIHR Clinical Lecturer and Specialist Registrar in Pediatric Dentistry, University of Leeds, Level 6/Worsley Building, Leeds, LS2 9LU UK; 2 School of Dentistry, Jordan University of Science and Technology, Jordan, UK

**Keywords:** Early childhood caries, Mother perception, Mother knowledge, Barriers seeking dental treatment

## Abstract

**Purpose:**

To assess maternal knowledge, attitudes and beliefs of Early Childhood Caries (ECC) risk factors and to determine barriers in seeking dental treatment among children with ECC.

**Methods:**

A total of 600 mothers of healthy children, aged 3–5 years, with ECC attending maternity and child health centres in Jordan completed a questionnaire using face-to-face interviews. ECC was diagnosed clinically by the chief investigator, based on the diagnostic criteria suggested by American Academy of Paediatric Dentistry (AAPD), and caries was recorded using dmft index.

**Results:**

The majority of the children had poor oral health status (99.2%) with a dmft index of 6.04 (± 1.2). Less than third (25.7%) of the mothers believed that their child had poor oral health with more than half (53.3%) not being aware that their child had dental caries. Most of the mothers had poor knowledge, attitudes and beliefs regarding their children’s oral health (82%). Participants` oral health knowledge was significantly associated with mothers` level of education and profession (*p* < 0.05). In addition, there was delay in seeking dental treatment for their children by the majority (65.9%) of mothers. Maternal profession, family income and time needed to reach a nearby health centre were found to be significant barriers in seeking dental treatment (*p* < 0.05).

**Conclusion:**

The majority of the mothers of children in Jordan with ECC had poor knowledge about their children’s oral health status. Moreover, seeking dental treatment was delayed by a large number of mothers of children with ECC.

## Introduction

Early childhood caries (ECC) is a severe form of dental caries affecting children under the age of six. It is characterised by affecting the smooth surfaces of primary teeth soon after their eruption. In addition, It progresses rapidly and results in detrimental impact on the dentition and child`s quality of life (AAPD 2014; Anil and Anand 2017; BaniHani et al. 2018).

Despite a general decline in caries prevalence, ECC remains a major health problem in infants and toddlers (Ismail and Sohn 1999; Grauwe and Martens 2004; Retnakumari and Cyriac 2012; Mahesh et al. 2013; Javed et al. 2017). ECC has a complex and multifactorial aetiology. It results from a complex interaction between the use of sweetened pacifiers, nursing on demand, neglected oral hygiene, increased streptococcus Mutans count, carer education and dental knowledge, family structure as well as socio-economic status (Simratvir et al. 2009; Congiu et al. 2014).

Infants and preschool children are particularly vulnerable to dental caries. Carers play an important role in the well-being of young children as they are the primary decision makers in terms of their children's health-related behaviours and health care. Therefore, exploring their knowledge, attitude, beliefs and barriers facing them in seeking oral health care for their children are important considerations in an attempt to make improvements in children`s well-being (Shivaprakash et al. 2009; Chhabra and Chhabra 2012; Mahesh et al. 2013). Kay and Locker suggested that an understanding of mother’s knowledge of their children oral health issues is crucial to modify their behaviour and encourage good health promotion (Kay and Locker 1996).

Mothers' dental awareness and barriers to accessing oral healthcare are important contributing factors to ECC but is not adequately addressed in literature reviews. Shivaprakash et al. (2009) investigated mother`s knowledge and awareness of their infants` oral healthcare in two socio-economic classes; rural and urban areas in India. They found maternal knowledge was inadequate irrespective of the locality. Their finding is in agreement with Chhabra and Chhabra (2012) who believed that maternal knowledge and attitudes regarding the importance of their children dental health needed to be improved. In Jordan, there is lack of information about mothers` knowledge, attitude and believes regarding their children’s oral health. Rajab et al. (2002) found a discrepancy between maternal dental knowledge and oral health care practices in Jordan. The results indicated that children and mothers` attitudes toward dental care needed to be improved.

In addition, ECC remains untreated in some children partially due to inadequate access and utilisation of health care services. Studies from different countries indicated that barriers to accessing oral healthcare were many, interactive and vary in different communities (Rajab et al. 2002; Al-Omiri et al. 2006). Barriers included perceptions of need, financial cost, occupational stress and lack of access. Al-Omiri et al. (2006) reported that the main barriers for regular dental attendance in Jordan were “treatment not needed” as well as “cost”. Other barriers were: fear from dental treatment and lack of parental encouragement to visit the dentist regularly. Rajab et al. (2002) considered the shortage in the number of paediatric dentists to be the most important barrier in seeking dental care among children and their families.

This study investigated mothers` attitudes, knowledge and beliefs of ECC risk factors and their utilisation of dental health services available in Jordan. It highlighted the role of mother`s perception of their children in developing ECC and attempted to determine barriers against utilising oral health services among children with ECC.

## Materials and methods

### Study population and ethics

In total, 650 mothers of medically healthy children diagnosed with ECC attending maternity and child health centres (MCHCs) for regular check-up and vaccinations in Amman (375 children) and Irbid (275 children) were approached to participate in the study, representing the Northern and Middle parts of Jordan, respectively. The Ministry of Health and the Institutional Review Board (IRB) at the Jordan University of Science and Technology approved the study. Participant Information Sheet (PIS) was given as well as written consent was obtained from all participants prior to the study.

Participants were included in the study if they met the following criteria: child with no significant health problem (ASA Physical Status 1 and 2) and had ECC on dental examination at the time of the study; mother able to give consent and speaks the Arabic language.

### Diagnostic Criteria of ECC and clinical examination

ECC was diagnosed clinically based on the diagnostic criteria suggested by the American Academy of Pediatric Dentistry (AAPD) (2008). Children were considered to have ECC if they had one or more decayed (non-cavitated or cavitated lesion), missing (due to caries) or filled tooth surfaces in any primary tooth in a child 71 months of age or younger. In addition, dmft index was used to record caries for each primary tooth present. The eruption dates of primary teeth were taken into consideration when teeth were examined for ECC.

Dental caries examination was conducted in a systematic manner using individually wrapped, sterilised dental mirrors and tongue depressors by a well-trained and calibrated single examiner (AB). The information was recorded on a prepared examination section in each questionnaire by a research assistant.

### The Questionnaire

Data on mother’s knowledge, attitudes and beliefs as well as barriers against seeking dental treatment were collected by the use of a structured questionnaire developed by the researcher (AB). The questionnaire was first designed in English and then translated into Arabic by a professional translator.

The questionnaire was piloted among a non-target sample of 20 mothers of children with ECC, none of the questions had to be modified. In addition, the reliability and validity of the questions were measured using test–retest and Cronbach’s Alpha. The participants completed the questionnaire through face-to-face interview.

The questionnaire comprised of 4 sections with total of 26 closed questions. It aimed to assess the following: (i) demographic details of the child and his mother (9 questions) including child gender, residence, mother’s age, mother’s education level (uneducated, high school degree and bachelor degree), mother`s field of work (professional when attended university or college, skilled when attended technical school, housewife when main occupation is caring for the family), monthly income of the family and type of house the family lives in, (ii) mother`s perceptions of their child`s oral health status (one question), (iii) mother`s attitudes and knowledge regarding oral health education and dietary habits (11 questions), and (iv) the availability and accessibility of health services and insurance (5 questions).

In the present study, mother`s knowledge regarding oral health education and dietary habits was assessed in Sect. 3 of the questionnaire and comprised of 11 questions. They were classified as ‘Good knowledge’, ‘Poor knowledge’ and ‘No knowledge’ according to the number of correct answers provided. ‘Good knowledge’ score was given if mother had 9 or more questions correct, 4–8 correct questions were given a ‘Poor knowledge’ score and those who answered less than 4 questions correctly were given the ‘No’ knowledge score.

In addition, delay in seeking dental treatment in the present study was predetermined as those children with ECC who had not visited the dentist in the past 6 months.

### Statistical analysis

Data were entered into SPSS (Statistical Package for the Social Sciences) version 22. Data were analysed using descriptive statistics, comparisons of means and test of association. Statistical analyses of association of mother`s perception with various categorical variables were performed using Chi-square. Probability values ≤ 0.05 were considered statistically significant. Intra-examiner reliability was calculated using kappa score and was found to equal 0.90. In addition, the reliability and internal consistency of the questionnaire were found 0.82 and 0.895, respectively.

## Results

### Socio-demographic characteristics of the study population

The socio-demographic characteristics of the study population are shown in Tables 1 and 2.

In total, 600 children and their mothers participated in the study providing a response rate of 92%. Children`s age ranged between 3 and 5 years (median = 3.95 ± 0.5) with slightly more than half females (*N* = 302, 50.3%). Mothers’ age ranged between those who were younger than 25 years (*N* = 54, 9.1%) and those who were over 40 years (*N* = 72, 12.0%). Among mothers, 78.8% (*N* = 473) had high school education, and the majority were housewives (*N* = 505, 84.2%) with only 14% (*N* = 84) having professional jobs. In addition, nearly two thirds (*N* = 371, 61.8%) of the families had only enough income to cover their monthly expenses, 34% (*N* = 204) were in debt, and a minority (*N* = 25, 4.2%) stated that they saved money from their monthly income.

**Table 1:** Socio-demographic characteristics of the study population (N = 600 pairs of mothers and children diagnosed with ECC)

Variable	Category	*N *(%)
Child gender	Males	298 (49.7%)
	Females	302 (50.3%)
Type of residence	Urban	586 (97.7)%
	Rural	14 (2.3%)
Residence	Amman	350 (58.3%)
	Irbid	250 (41.7%)
Maternal age	< 25 years	54 (9.1%)
	25–30 years	161 (26.8%)
	30–35 years	140 (23.3%)
	35–40 years	173 (28.8%)
	> 40 years	72 (12.0%)

**Table 2:** Educational and socio-economic characteristics of the study population (*N* = 600 pairs of mothers and children diagnosed with ECC)

Variable	Category	*N* (%)
Mother’s education level	Uneducated	21 (3.5)
	High school degree	473 (78.8)
	Bachelor degree	106 (17.7)
Mother’s occupation	Professional	84 (14.0)
	Skilled	11 (1.8)
	Housewife	505 (84.2)
Family income	Save monthly	25 (4.2)
	Income = monthly expenses	371 (61.8)
	In debt	204 (34.0)
House type	Owned	303 (50.5)
	Rented	297 (49.5)

### Actual and perceived oral health status of children with ECC

The majority (*N* = 550, 91.6%) of children in the present study had poor oral health status as assessed by the examiner (AB) with a median dmft index of 6.04 (± 1.2). On the contrary, less than one third (*N* = 154, 25.7%) of the mothers believed that their child had poor oral health with more than half (*N* = 320, 53.3%) not aware that their child had dental caries. A poor agreement (kappa = 0.155) was found between the mother`s perceived oral health status of the child and the actual oral health status.

### Maternal knowledge, attitude and beliefs of ECC risk factors

Maternal knowledge, attitudes and beliefs regarding ECC risk factors are summarised in Table 3. Almost two thirds of the mothers in the present study (*N* = 363, 60.5%) had received some dental education, mainly through the media (*N* = 208, 57.3%) and by the dentist (*N* = 155, 42.7%). In addition, 82% (*N* = 489) were found to have ‘poor knowledge’ of oral health issues concerning the aetiology and prevention of ECC, 11% (*N* = 69) had ‘no knowledge’ and only a minority (*N* = 42, 7%) had ‘good knowledge’.

**Table 3:** Description of the mothers’ attitudes, beliefs and knowledge of ECC risk factors (*N* = 600 pairs of mothers and children diagnosed with ECC)

Statement	True	False	I don’t know
Should avoid sharing food	237 (39.5%)	354 (59.0%)	9 (1.5%)
Baby teeth are important as the adult teeth	507 (84.5%)	65 (10.8%)	28 (4.7%)
Sweets can cause decay	552 (92.0%)	30 (5.0%)	18 (3.0%)
Soft drinks can cause decay	423 (70.5%)	115 (19.2%)	62 (10.3%)
Breast feeding can cause decay	108 (18.0%)	457 (76.2%)	35 (5.8%)
Should avoid sweet snacks	519 (86.5%)	24 (4.0%)	57 (9.5%)
Fluoride prevents decay	256 (42.7%)	53 (8.8%)	291 (48.5%)
Excess fluoride causes fluorosis	90 (15.0%)	87 (14.5%)	423 (70.5%)
Best time for weaning is one year old	103 (17.2%)	436 (72.7%)	61 (10.1%)
Teeth cleaning should start after eruption of first baby tooth	253 (42.1%)	271 (45.2%)	76 (12.7%)
Should start visiting the dentist during 6 months of primary tooth eruption	230 (38.3%)	292 (48.7%)	78 (13.0%)

The correlation between the participants’ demographic and educational characteristics with their knowledge, attitudes and beliefs was assessed in the current study. Only mothers’ level of education and profession had a statistic significant effect on the level of their oral health knowledge, attitudes and beliefs (*p* < 0.05). Mothers with bachelor degrees as well as professional mothers demonstrated ‘good knowledge’; whereas ‘no knowledge’ was more often seen among uneducated mothers and housewives. In addition, an attempt was made to correlate the mother knowledge score with the dmft index score of their children but no statistic significant difference was found (*p* = 0.122).

### Accessibility to Dental Health Services and factors influencing their use among the participants

Most of the mothers in the present study (*N* = 587, 97.8%) knew of a health centre providing dental treatment nearby with the majority (*N *= 572, 96.5%) required less than one hour travelling time to reach the health centre from their place of residence. Governmental health insurance including dental benefits was reported by 58% (*N* = 348), of the families (Table 4).

**Table 4:** Availability and accessibility of utilising dental health services among mothers of children with ECC (*N* = 600 pairs of mothers and children diagnosed with ECC)

Variable	Category	*N* (%)
Knowledge of dental health centre nearby?	Yes	587 (97.8)
	No	13 (2.2)
Time required reaching the destination?	< 1 hour	572 (96.5)
	> 1 hour	21 (3.5)
Health insurance including dental?	Yes	348 (58.0)
	No	252 (42.0)
Did your child ever visit a dentist?	Yes	354 (59.0)
	No	246 (41.0)
If yes, when was it?	> 6 months	218 (65.9)
	6–12 months	63 (19.0)
	< 6 months	50 (15.1)

In total, two thirds (*N* = 218, 65.9%) of the mothers reported that they had delayed in seeking dental treatment for their children (did not visit the dentist for more than 6 months). Maternal profession, family income, and time needed to reach a nearby health centre were found to affect the delay in seeking dental treatment significantly (*p* < 0.05). More delay was seen among the skilled mothers (*N* = 150, 68.8%), followed by housewives (*N* = 40, 18.4%) and professional mothers (*N* = 28, 12.8%). Families who had financial problems (income = outcome or in debt) exhibited more delay in seeking dental treatment (*N* = 140, 64%) as opposed to 36% (*N* = 78) of those with extra income. Interestingly, the shorter the time needed to reach a nearby Health Centre, one hour or less, the more the delay in seeking treatment (*p* < 0.003).

## Discussion

Maternal knowledge, attitudes and believes of ECC risk factors as well as barriers to accessing oral healthcare are important contributing factors to ECC but less addressed in the literature. Mothers’ perception has an important impact on children's oral health and oral health-related behaviours. In Jordan, 62% and 73% of the 4- and 5-year olds were found to have ECC with dmft values of 3.1 and 4.1, respectively (Gussy et al. 2008). The current study was a cross-sectional study with a sample of 600 pairs of mothers and children diagnosed with ECC attending MCHCs for check-up and vaccination. The study aimed to explore mothers` knowledge, attitudes and believes of ECC risk factors as well as to determine the factors affecting their utilisation of oral health services in Jordan.

Although most of the mothers in Jordan (84.5%) believed that primary teeth were as important as permanent teeth, the majority (82%) were found to have ‘poor knowledge’ of oral health issues concerning the aetiology and prevention of ECC and only 7% of the mothers demonstrated ‘good knowledge’. Over 60% of mothers in the current study believed that sharing food with their children was acceptable. This finding was consistent with previous studies where nearly half of the mothers had sometimes shared food with their children (Blinkhorn 1981; Shivaprakash et al. 2009). As for weaning, only 17.2% of mothers agreed with the idea that the best time for weaning was at one year of age with the majority (76.2%) reporting that breast feeding had no effect on the development of ECC. A higher proportion was reported in a previous study carried out in India (Shivaprakash et al. 2009).

Fluoride is one of the most effective protective measures in preventing dental caries. A consistently weak knowledge regarding fluoride’s role in caries prevention was observed among the participants in the present study as less than half of mothers (42.7%) believed that fluoride can prevent tooth decay, while almost half of the mothers did not know anything about fluoride’s role in caries prevention (48.5%). Moreover, the majority of mothers in the present study failed to correctly point out a clear relation between excess fluoride and tooth discolouration (70.5%). These finding reflect the fact that comprehensive preventive programmes and education regarding fluoride`s role in caries prevention among the population are still lacking in Jordan; hence, more dental health education regarding fluoride is needed.

An oral health consultation visit within 6 months from eruption of the first tooth and no later than 12 months of age is recommended by AAPD to educate carers and provide anticipating guidance for prevention of dental disease (AAPD 2008). Only one third (38.3%) of the mothers in the present study were aware of this recommendation, and a high proportion of them did not clean their child’s mouth after the eruption of the first primary tooth (58%). A similar finding had been reported previously by Shivaprakash et al. (2009). However, the findings of Gussy et al. (2008) have disagreed with the present study findings, and indicated a higher dental awareness of mothers living in Australia.

On the other side, the majority of the mothers believed that sweetened pacifiers and exposure to sweetened liquids in bottles at night were related to ECC (92.0% and 70.5%, respectively). In addition, most of the mothers (86.5%) agreed that drinks in-between meals should be restricted to water or plain milk only.

Mothers’ knowledge, attitudes, and beliefs of ECC risk factors ware associated with their socio-demographic characteristics (Williams et al. 2002; Amschler 2003). Socio-demographic characteristics including ethnicity, parental deprivation, and educational status have been found to affect dental health knowledge and attitude. In the present study, maternal level of education as well as mother’s profession had a statistical significant effect on the level of their oral health knowledge, attitudes and beliefs. ‘No knowledge’ was seen mainly among the uneducated mothers and housewives, while mothers with a bachelor degree as well as professional jobs had the highest proportion of ‘good knowledge’; this finding was in agreement with the study of Williams et al. (2002). A general improved level of mothers` education and profession may mean that mothers are more able to access appropriate sources of information and understand the information more fully than mothers with lower educational levels. The main message about dental health did not seem to be understood by those most in need of information, for example most participants were aware that sugar causes dental caries; however, frequency of sugar uptake had not been given any attention. Therefore, simple, clear and consistent message from oral health professionals are needed to ensure that the information given is in a form that is readily understood by mothers and can be related to every day practice.

In the present study, the level of agreement was low between the mother’s perceived oral health status of their child and the actual oral health status as noted by the chief investigator. This is due to the fact that the majority of mothers in this sample had ‘poor knowledge’ regarding the oral health of their children.

A significant proportion (65.9%) of children had delayed seeking dental care, and this can be contributed to the fact that the majority of the mothers in Jordan had poor knowledge and incorrect perceptions regarding their children`s oral health status; therefore there is a need to educate parents regarding the importance of oral health care and regular check-ups.

The utilisation of dental health services in the present study was significantly affected by several factors, namely: mother’s profession, family income, and time needed to reach a nearby health centre. Such information is inadequately addressed in the literature. The majority of the delay in seeking dental treatment was among the skilled (81.8%) followed by housewives (65.1%) and professional mothers (52.4%). Skilled mothers usually have no free time to spend with their children and are less educated mostly to high school education. While, housewives spend most of their time at home not exposed to a wider variety of experience and knowledge resources. Participants who had financial problems (income = outcome or in debt) exhibited delay in seeking dental treatment (67.7% and 59.8%, respectively) compared with those of a better financial condition. The fact that financial status of the family was a barrier to dental care had been reported previously in the literature (Agili et al. 2005; McBroome et al. 2005; Al-Omiri et al. 2006). This finding contributes to the point that disadvantaged children are more likely to have a higher proportion of caries and more unmet treatment needs than their higher-income counterparts because of the high cost of dental treatment (Kikwilu et al. 2008). People with low socio-economic status face barriers to dental care, most importantly are: high cost of dental treatment, lack of demand to dental care, poor distribution of paediatric dentists, lower rate of insurance coverage, difficulty in transportation, and less availability of dental educational programmes (Rajab et al. 2002). Position and location of dental health services play an important role in dental health services utilisation. Surprisingly in the current study, families who live closer to a dental health centre exhibited more delay in seeking dental treatment compared to those who lived further away and required more than one hour to get there. This was in contrast with Kikwilu et al. (2008) who reported that distance was a barrier and caused delay in obtaining treatment. Jacob and Plamping (1989) also believed that patients usually use health services which are within a 6 miles (10 kms) radius of their homes, work or schools and people in rural areas find arranging for transportation a barrier for seeking oral health care. The possible explanation for this could be that mothers living nearby a health centre can assume that they can seek treatment anytime and can attend even in cases of emergency because of its convenience. Whereas those families living far away from a dental care centre where seeking treatment requires advance planning and moreover perhaps due to fear of possible dental emergency or pain at night, mothers were inclined to book appointments, organise transport and attend for dental treatment.

This study is not without limitations. The sample was a convenient sample, including only mothers and healthy children who were diagnosed with ECC attending MCHCs in two cities in Jordan; therefore, generalisation of the results may not be appropriate. In addition, the study utilised a self-reported questionnaire which depended on mother’s subjectivity; this could contribute to a response bias. Moreover, the study results were discussed with regards to the mother`s education and occupation without any reference to factors related to the father. This was for the reason that mothers in Jordan are often the main carers for their children.

## Conclusion

The majority of the mothers of children diagnosed with ECC in Jordan had poor knowledge regarding their children’s oral health status. Although most mothers believed that primary teeth were as important as permanent teeth, most had poor knowledge of the aetiology and prevention of dental caries. In addition, seeking dental treatment was delayed in the majority of mothers of children with ECC in Jordan; skilled mothers, families with financial problems, and decreased distance from a dental centre were barriers facing dental treatment seeking among mothers in Jordan.
